# Transcranial Doppler Ultrasonography-Related Research in the Caribbean Region

**DOI:** 10.7759/cureus.35147

**Published:** 2023-02-18

**Authors:** Kesava Mandalaneni, Priyanka Venkatapathappa, Sarah Koshy, Gabrielle Walcott-Bedeau, Vajinder Singh

**Affiliations:** 1 Department of Neuroscience, St. George's University School of Medicine, St. George's, GRD; 2 Department of University Health Services, St. George's University, St. George's, GRD; 3 Department of Pathology, St. George's University School of Medicine, St. George's, GRD

**Keywords:** jamaica, brain death, hydroxyurea, vasospasm, stroke, sickle cell disease, transcranial doppler, grenada, west indies, caribbean

## Abstract

Transcranial Doppler (TCD) ultrasonography is a non-invasive ultrasound technique that uses high-frequency sound waves to measure blood flow velocities in the cerebral vasculature. This review analyzes TCD research in the Caribbean region using a bibliometric analysis of 29 articles from PubMed. The articles were analyzed using Microsoft Excel 2016 and the VOSviewer software (Van Eck and Waltman, Leiden University, Centre for Science and Technology Studies (CWTS), www.vosviewer.com) and characterized various aspects of TCD research, including countries, research themes, authorship, journals, affiliations, and keywords. The majority of the 29 publications came from Cuba (38%), followed by the French West Indies (22%) and Jamaica (20%). Most TCD research focused on sickle cell disease (SCD), accounting for 45% of the studies, followed by 21% of articles on vasospasm and subarachnoid hemorrhage. The use of TCD in brain death and neuro-intensive care was also explored, constituting 17% of the studies. Alternative TCD-monitored treatment options for SCD, such as stem cell transplantation and hydroxyurea, were also frequently investigated. The most productive institutions were Hospital Clínico-Quirúrgico Hermanos Ameijeiras in Havana, Cuba, the Sickle Cell Unit at the University of West Indies (UWI) Mona in Jamaica, the Medical-Surgical Research Center (CIMEQ) in Havana, Cuba, and the SCD Reference Center in Guadeloupe and Martinique in the French West Indies. TCD has been identified as a cost-effective tool for real-time monitoring of cerebral blood flow in many clinical settings, including stroke and SCD, which are prevalent in the Caribbean. Although there is an increase in the trend of using TCD for neuromonitoring in the Caribbean, gaps still exist. Capacity-building initiatives, such as training programs for healthcare providers and the development of local TCD research networks, can improve access to TCD in resource-constrained settings to treat and neuromonitor patients cost-effectively.

## Introduction and background

Transcranial Doppler (TCD) ultrasonography is a non-invasive ultrasound technique that uses high-frequency sound waves to measure blood flow velocities in the cerebral vasculature. Rune Aaslid introduced TCD ultrasonography for detecting blood flow in the basal intracranial vasculature, while Mark Moehring developed techniques to precisely measure the intensity and direction of blood flow in the intracranial space [1]. Its diagnostic and neuromonitoring uses frequently include detecting blood flow in the basal intracranial vasculature, detecting a right-to-left shunt, detecting spontaneous embolic signals, measuring vasomotor reactivity, monitoring vasospastic complications following subarachnoid hemorrhage (SAH), measuring intracranial pressure in various encephalopathies, as a supplementary test in confirming brain death, non-invasive bedside monitoring of cerebral hemodynamic patterns during recanalization in an acute ischemic stroke (AIS) setting, and identifying children at high risk of experiencing their first-ever stroke due to Hemoglobin SS (HbSS) and intervening with transfusion or hydroxyurea treatment [2,3].

Considering the cost-effectiveness of TCD for monitoring cerebral blood flow in real-time in various clinical settings, most notably in stroke and sickle cell disease (SCD), this study aimed to analyze how TCD has been used in the Caribbean region and review TCD-related research trends and outputs.

## Review

Methods

Data Source and Search Strategy

All analyzed articles were retrieved from the PubMed database of the National Center for Biotechnology Information (NCBI). The search was conducted on the 12th of January 2023, and all the articles till that date were included. In PubMed, publications were identified by searching for the terms ("barbados"[All Fields] OR "trinidad and tobago"[All Fields] OR "guyana"[All Fields] OR "suriname"[All Fields] OR "guiana"[All Fields] OR "guadeloupe"[All Fields] OR "martinique"[All Fields] OR "grenada"[All Fields] OR "dominican republic"[All Fields] OR "dominica"[All Fields] OR "saint lucia"[All Fields] OR "saint kitts and nevis"[All Fields] OR "aruba"[All Fields] OR ("saint vincent and the grenadines"[MeSH Terms] OR ("saint"[All Fields] AND "vincent"[All Fields] AND "grenadines"[All Fields]) OR "saint vincent and the grenadines"[All Fields]) OR "bahamas"[All Fields] OR "jamaica"[All Fields] OR "west indies"[All Fields] OR "french west indies"[All Fields] OR "caribbean"[All Fields] OR "cuba"[All Fields]) AND ("transcranial doppler"[All Fields] OR "tcd"[All Fields]) in the search field.

Inclusion Criteria

All articles from the Caribbean focused on TCD were included, at least one researcher in the publication should be representing a Caribbean Institution, and there were no restrictions regarding language or country of publication and types of articles.

Exclusion Criteria

Duplicates, not related to TCD.

**Figure 1:**
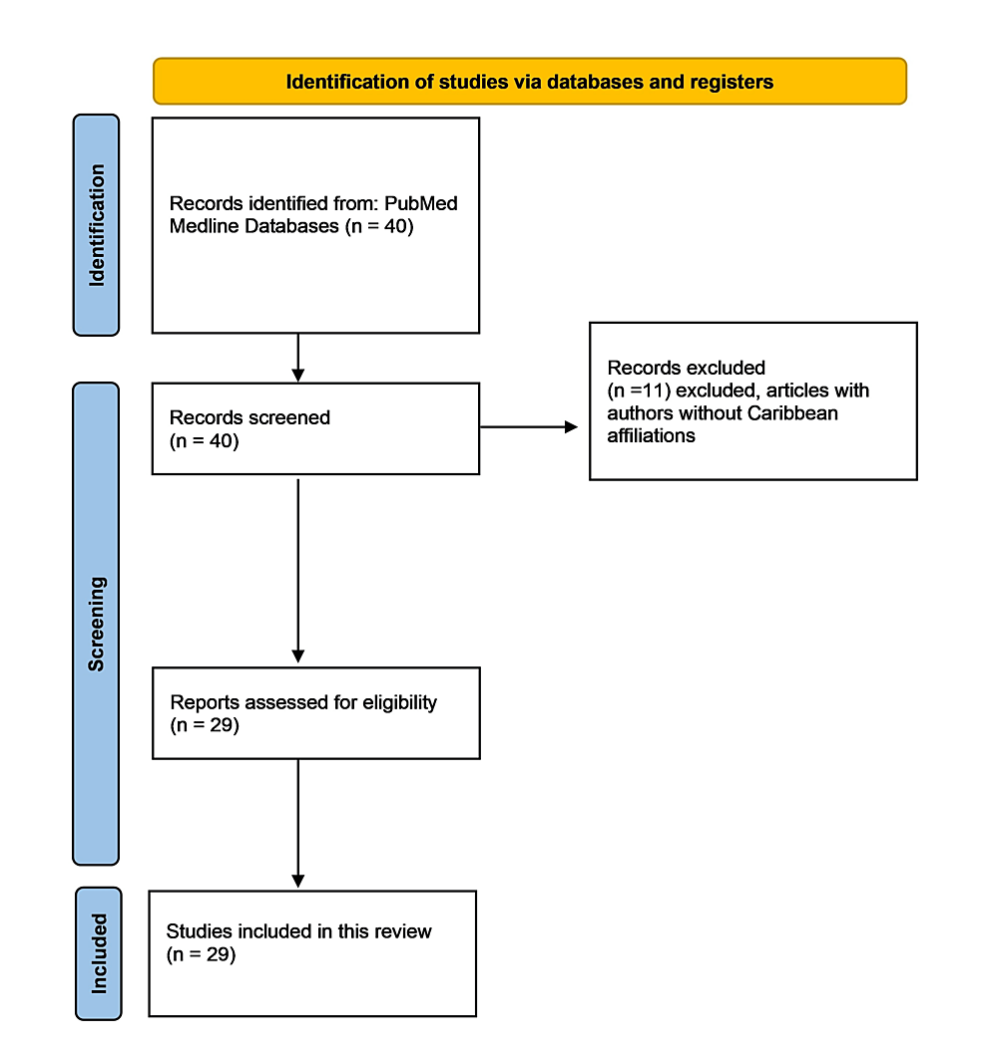
PRISMA flowchart of the review for Caribbean TCD research output The screening performed for this literature review follows guidelines in the PRISMA statement Image credit: This figure was created by the corresponding author (KM) using a PRISMA template [4]. PRISMA: Preferred Reporting Items for Systematic Reviews and Meta-Analyses; TCD: Transcranial Doppler

Three reviewers (KM, PV, VS) independently performed article screening. In case of conflict, it was resolved with discussion among the reviewers. Full articles were obtained whenever possible, and references were reviewed for appropriate citations. The selection process of articles is depicted in Figure 1. An analysis of various elements was carried out using Microsoft Excel and VOSviewer (Van Eck and Waltman, Leiden University, Centre for Science and Technology Studies (CWTS), www.vosviewer.com), including the number of publications per country, countries that were most active in conducting patient-related original research, most prolific researchers, types of research, themes explored in TCD research, and journals in which TCD research from the Caribbean was published.

Results and analysis

The study included a total of 29 publications. Out of the 40 initial search results, 11 articles were not included due to not meeting the required criteria. The VOSviewer software was utilized to analyze and visualize the published articles' tags.

Number of publications per country

**Figure 2:**
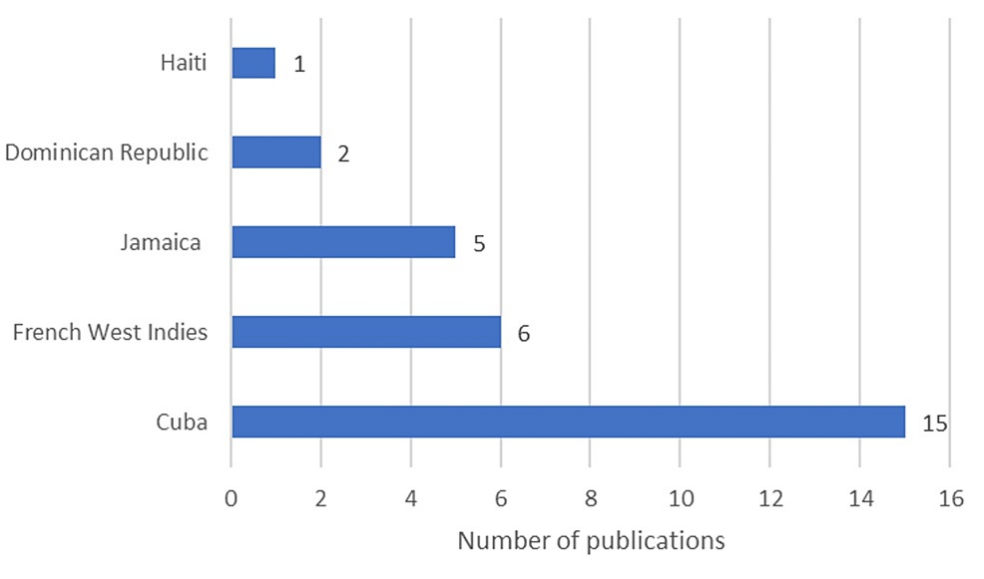
Total number of publications per country in TCD research Image credit: This figure was created by the corresponding author (KM). TCD: Transcranial Doppler

**Figure 3:**
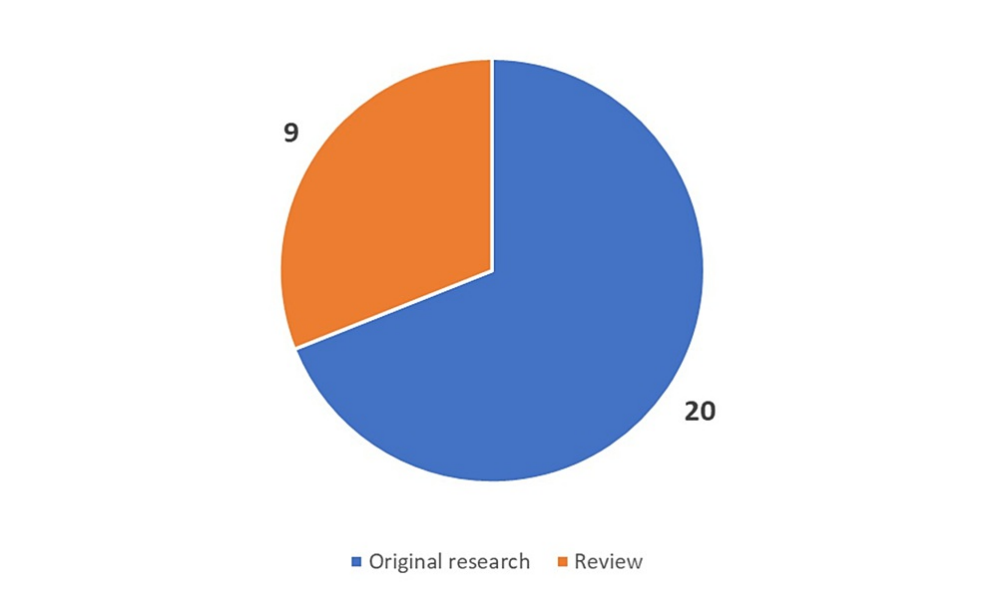
Types of research articles in Caribbean TCD research This figure was created by the corresponding author (KM). TCD: Transcranial Doppler

**Figure 4:**
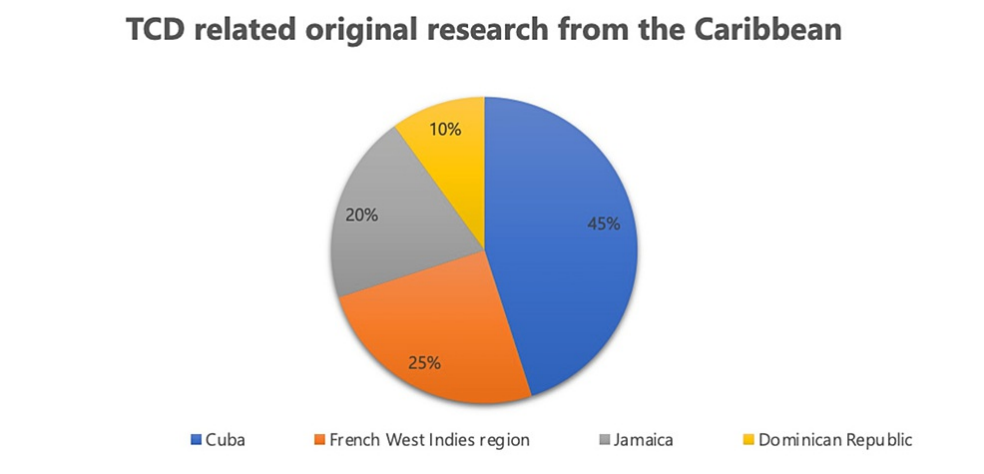
Country-wise TCD-related original research from the Caribbean Image credit: This figure was created by the corresponding author (KM). TCD: Transcranial Doppler

Of the 29 publications, the majority came from Cuba (38%), followed by the French West Indies (22%), Jamaica (20%), and other countries such as Haiti and the Dominican Republic also contributed to the TCD research (Figure 2). Out of the 29 articles, 19 (62%) were country-specific original research studies conducted in various Caribbean countries, with the largest contribution coming from Cuba (9/19), followed by the French West Indies (5/19), Jamaica (4/19), and the Dominican Republic (2/19) (Figure 3 and Figure 4).

Analysis of research themes

**Figure 5:**
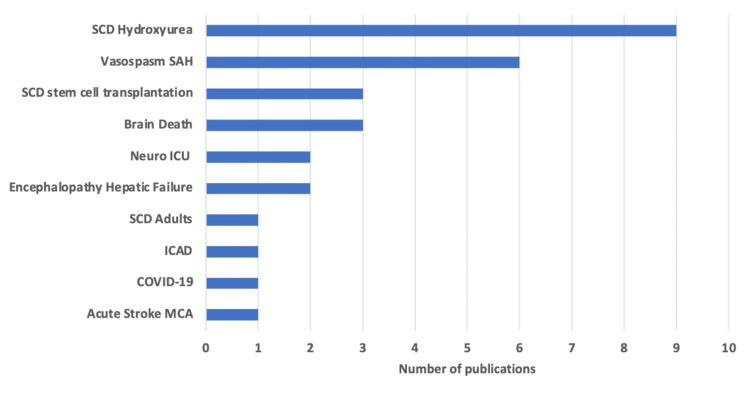
Thematic areas of TCD research Image credit: This figure was created by the corresponding author (KM). TCD: Transcranial Doppler; SCD: sickle cell disease; SAH: subarachnoid hemorrhage; ICU: intensive care unit; ICAD: intracranial atherosclerotic disease; MCA: middle cerebral artery

The majority of TCD research was centered around SCD accounting for 45% of the studies. A significant proportion of studies also focused on vasospasm and SAH, accounting for 21% of the studies. TCD usage in the areas of brain death and neurointensive care unit was also explored, constituting 17% of the studies (Figure 5). Within the SCD theme, researchers frequently looked into alternative treatment options like stem cell transplantation (SCT) and hydroxyurea, which don't involve blood transfusions. There was also a notable study that examined the use of TCD in monitoring patients who had recovered from COVID-19.

Analysis of journals

**Figure 6:**
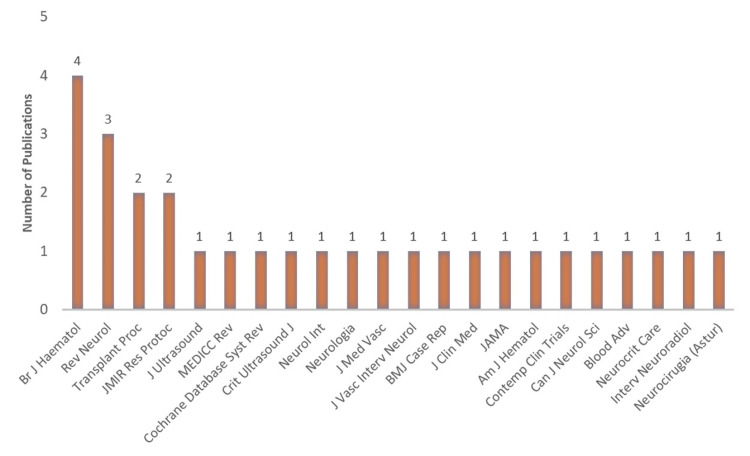
Journals in which TCD research from the Caribbean was published British journal of haematology, Revista de neurologia, Transplantation proceedings. JMIR research protocols, Journal of ultrasound, MEDICC review, The Cochrane database of systematic reviews, Critical ultrasound journal, Neurology international, Neurologia, Journal de medecine vasculaire, Journal of vascular and interventional neurology, BMJ case reports, Journal of clinical medicine, JAMA, American journal of hematology, Contemporary clinical trials, The Canadian journal of neurological sciences. Le journal canadien des sciences neurologiques, Blood advances, Neurocritical care, Interventional neuroradiology, Journal of peritherapeutic neuroradiology, surgical procedures and related neurosciences, and Neurocirugia Image credit: This figure was created by the corresponding author (KM). TCD: Transcranial Doppler

Figure 6 represents journals in which TCD research associated with the Caribbean was published. They include journals like the *British journal of hematology*, *Revista de neurologia*, *Transplantation proceedings,* and *JMIR research protocols*. Notably, there is an update to the Cochrane database.

Author analysis (first author/last author and institutional affiliations)

**Figure 7:**
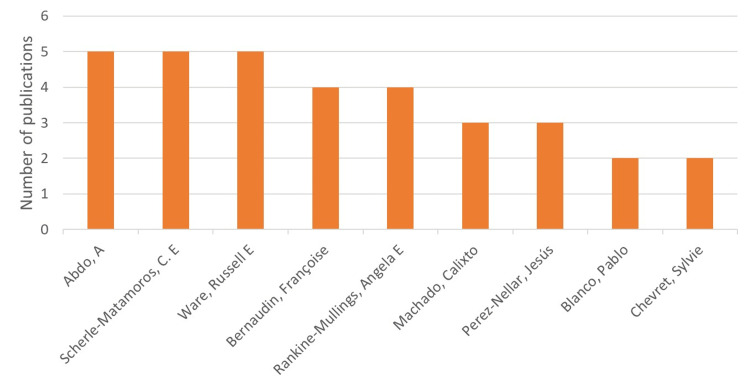
Most prolific authors Image credit: This figure was created by the corresponding author (KM).

The top contributing authors for TCD-related research are depicted in Figure 7. Of the 29 articles, 21 were led by a Caribbean researcher (with at least one of the first or last authors being from the Caribbean region).

**Figure 8:**
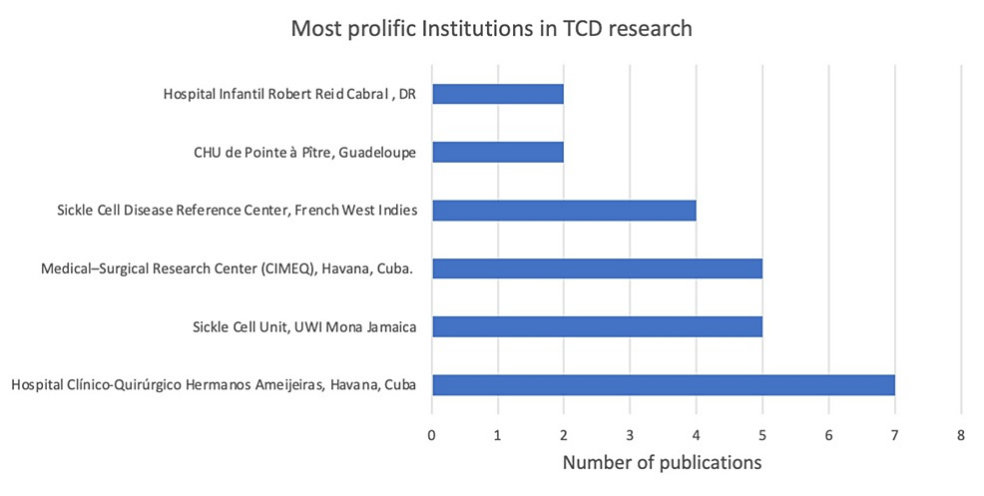
Most prolific institutions in TCD research Image credit: This figure was created by the corresponding author (KM). TCD: Transcranial Doppler

**Figure 9:**
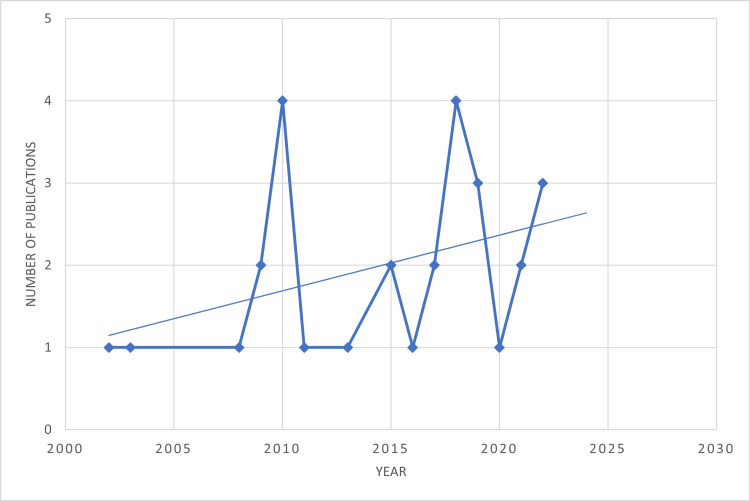
Upward trend in TCD research in the Caribbean Image credit: This figure was created by the corresponding author (KM). TCD: Transcranial Doppler

An analysis of institutional research output revealed that the most productive institutions were Hospital Clínico-Quirúrgico Hermanos Ameijeiras in Havana, Cuba, the Sickle Cell Unit at the University of West Indies (UWI) Mona in Jamaica, the Medical-Surgical Research Center (CIMEQ) in Havana, Cuba, and the SCD Reference Center in Guadeloupe and Martinique in the French West Indies. A general upward trend has been seen in the TCD research in the Caribbean (Figures 7-9).

Discussion

The Caribbean researchers utilized TCD in a variety of medical contexts, including SCD, monitoring for vasospasm in SAH, hepatic failure, as a follow-up of COVID-19 patients, and in the context of persistent vegetative state (PVS) and brain death. A summary of the conditions and the TCD parameters used in each of these contexts by the Caribbean researchers is discussed in the following paragraphs.

1. Sickle Cell Disease

SCD is a prevalent genetic condition among the Afro-Caribbean population in the Caribbean. Children with SCD, particularly those with SS/SB° genotypes, experience severe complications, with stroke being the most severe and having a risk of 11% by age 18 [1,5,6]. In these children, the underlying mechanism of an ischemic stroke is intracranial cerebrovascular steno-occlusion, caused by intravascular sickling and sludging [7]. Nevertheless, the incidence of stroke in these patients can be diminished through chronic transfusion therapy or by treatments such as hydroxyurea or SCT.

Use of TCD in SCD: TCD ultrasound is considered an effective and gold-standard method in screening and diagnosing cerebrovasculopathy in children with SCD. TCD-measured flow velocities in the intracranial arteries help identify individuals at a greater risk of stroke, allowing for prompt interventions. TCD screening in children with SCD for cerebrovasculopathy is done according to the stroke prevention in SCD (STOP) trial protocol where specific TCD parameters like the time-averaged mean of the maximum (TAMM) velocities of the distal internal carotid artery (dICA) and middle cerebral artery (MCA) is used for quantifying risk staging: Normal: less than 170 cm/s, Conditional: between 170-200 cm/s, Abnormal: greater than 200 cm/s [8,9].

**Figure 10:**
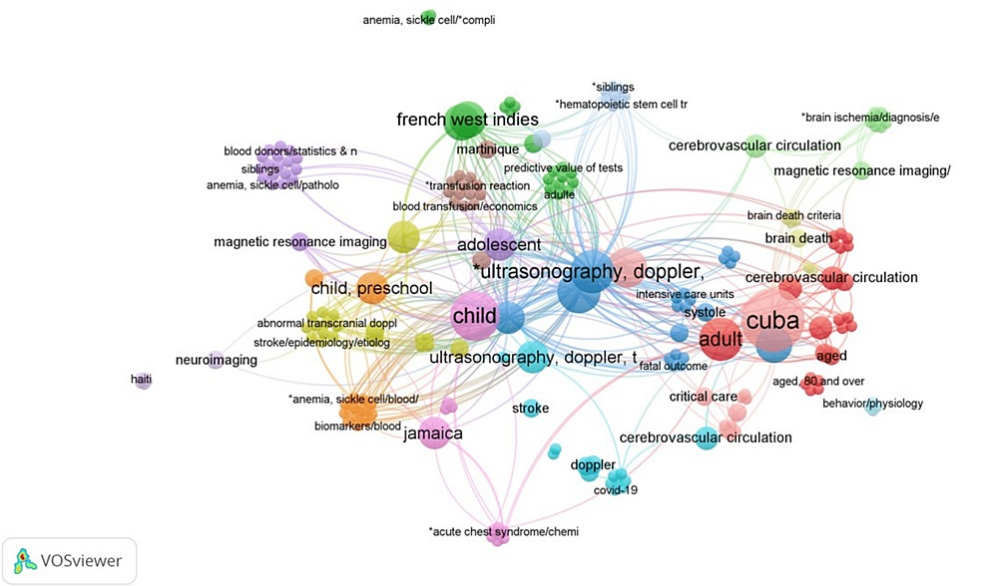
VOSviewer overlay visualization of tag analysis of the published articles Image credit: This figure was created by the corresponding author (KM) using the software VOSviewer based on the tags of the articles retrieved in the results. Analysis of the tags clearly shows that the most active countries in the TCD research are Cuba, French West Indies, and Jamaica. An interesting discovery in this tag analysis is the heightened focus on TCD-SCD research in territories with a predominantly Afro-Caribbean population, such as Jamaica and the French Caribbean, compared to other territories with a mixed or predominantly white population like Cuba. TCD: Transcranial Doppler; SCD: sickle cell disease

TCD-SCD-related research in the Caribbean: SCD is the most prevalent topic in the form of reviews and original research from the region. An intriguing finding is that most of the TCD-SCD research came from territories with a predominantly Afro-Caribbean population, such as Jamaica and the French Caribbean, as opposed to other territories with a mixed or predominantly white population like Cuba (Figure 10). The value of TCD screening in treating children with SCD is demonstrated by a national evaluation of SCD care in France and its Caribbean territories. The study found that there was a low risk of severe complications due to the effective TCD screening program and comprehensive healthcare system, including widespread vaccination coverage [10].

Chronic transfusions are used as a therapeutic intervention in children diagnosed with SCD to reduce high TCD velocities, which serve as a surrogate marker for the onset of ischemic stroke. Nevertheless, this treatment approach is not curative and poses the risk of adverse events, such as alloimmunization and iron overload. The requirement for ongoing transfusions for the primary prevention of strokes makes them unfeasible in countries with limited resources. Notable updates by Jamaican researchers to the Cochrane database emphasized the efficacy of hydroxyurea in primary stroke prevention in at-risk SCD patients by maintaining TCD velocities [11,12].

Most of the original TCD-SCD-related research in the region was derived from clinical trials that assessed non-transfusion therapies, including hydroxyurea (EXTEND, SACRED, and SCATE) or hematopoietic SCT (DREPAGREFFE).

According to the findings of the EXpanding Treatment for Existing Neurological Disease (ExTEND) trial conducted in Jamaica, administration of hydroxyurea up to the maximum tolerated dose (MTD) consistently resulted in decreased TCD velocities among individuals exhibiting abnormal or conditional velocities, thereby reducing the risk of a first-ever stroke. The trial underscored the significance of implementing a TCD screening program in conjunction with hydroxyurea therapy to decrease the impact of SCD and strokes in resource-constrained settings [13,14]. The study was conducted in partnership with the Cincinnati Children's Hospital Medical Center, which also collaborated with researchers from the Dominican Republic on a similar clinical trial, Stroke Avoidance for Children in REpública Dominicana (SACRED), on the efficacy of hydroxyurea in reducing TCD velocities [15]. Hematopoietic SCT offers the potential for stopping transfusion therapy, but its benefits have not been established. The DREPAGREFFE trial, a multicenter study conducted in France and the French Caribbean, sheds light on the safety, efficacy, and effectiveness of SCT as a treatment option for SCD patients. The trial results showed a reduction in TCD velocities, reflecting improved stenosis prevention, and better outcomes for patients who underwent SCT compared to those receiving standard treatment with chronic transfusions [16,17].

In the context of treatment to reduce cerebrovascular stenosis in children with SCD, regardless of the chosen method, be it transfusion, transplantation, or treatment with hydroxyurea, the use of TCD remains a crucial prerequisite in neuromonitoring and guiding the physician.

2. SAH-related Vasospasm

Vasospasm of the arteries is the most important complication of SAH that affects over 25% of SAH patients and may lead to a delayed ischemic deficit (DID) [18]. Digital Subtraction Angiography (DSA) is the most reliable method for diagnosing vasospasm in patients with aneurysmal SAH. In addition to clinical examination, TCD has also been considered a better option for monitoring and detecting vasospasm and delayed cerebral ischemia [19].

TCD in SAH-related vasospasm: TCD's usefulness in SAH is due to its ability to continuously monitor cerebral hemodynamics in the intracranial arteries over a long period of time. Most importantly, TCD renders it possible to detect the onset of vasospasm even before it becomes clinically and symptomatically apparent, track its progression, determine the severity of vasospasm in patients, assess the effectiveness of treatments, and identify patients in need of further endovascular interventions [20].

A rise in mean cerebral blood flow velocity indicates vasospasm in major cerebral vessels. The Therapeutics and Technology Assessment Subcommittee of the American Academy of Neurology states that TCD helps detect vasospasm after spontaneous SAH, which was validated in a study conducted on 89 patients with non-traumatic SAH. Monitoring with TCD proved useful for diagnosing cerebral vasospasm. The results showed that overall precision, sensitivity, and a positive predictive value of TCD monitoring were effective [1,21].

In multiple studies, Scherle-Matamoros et al. validated the predictive abilities of early TCD evaluation of mean flow velocities (MFVs) in the MCA and anterior cerebral artery (ACA) in the development of symptomatic vasospasm (sVSP) in patients with acute SAH. They also observed that cognitive and behavioral (53%) and focal neurological deficits (26%) were the most frequent symptomatic manifestations based on TCD vasospastic parameters [22,23].

3. Hepatic Failure and TCD

The outcome of patients with fulminant hepatic failure (FHF) may be impacted by cerebral edema and intracranial hypertension. To evaluate intracranial hypertension, cerebral hemodynamic patterns, and brain function in these patients, TCD parameters like pulsatility index (PI) and MFVs are commonly used. In their studies, Abdo et al. found that patients with FHF exhibited a predominant pattern of cerebral hypoperfusion, characterized by decreased mean velocities and elevated pulsatility index, indicating increased resistance. These findings highlight the utility of TCD in predicting prognosis and guiding therapeutic interventions for patients with FHF [24,25].

4. COVID-19 and TCD

Severe acute respiratory syndrome coronavirus 2 (SARS-CoV-2) infection can cause endothelial injury and microvascular damage. TCD ultrasound can be utilized to determine the state of the cerebral hemodynamic reserve, which could provide insight into any cerebrovascular endothelial dysfunction. Despite its potential, the application of this technique in patients with COVID-19 is limited, and it is not currently a part of the COVID-19 action and follow-up guidelines [26].

Abdo-Cuza et al. observed that patients who recovered from SARS-CoV-2 infection showed decreased cerebral hemodynamic reserve and breath-holding index, regardless of the disease's clinical severity or presence of neurological symptoms [26]. These abnormalities may be linked to the endothelial damage caused by COVID-19, making it important to consider TCD as a useful tool in evaluating and following follow-up protocols for patients with COVID-19.

5. TCD Uses in Brain Death and PVS

Brain death refers to the complete and permanent loss of all brain function, including that of the brainstem. PVS also referred to as unresponsive wakefulness syndrome (UWS) is a condition often brought about by severe brain injuries, such as traumatic brain injury, stroke, or anoxia. This condition is caused by diffuse cortical injury, which results in a loss of consciousness and awareness, although the brainstem remains functional.

The diagnosis of brain death is established through serial clinical evaluation that assesses brain and brainstem functions and uses confirmatory tests, such as TCD ultrasound and electroencephalogram (EEG). The clinical evaluation using various provocative measures is considered the gold standard for diagnosing brain death. TCD can assist in diagnosing cerebral circulatory arrest and can help to confirm the absence of brain perfusion through its parameters, such as the oscillating flow pattern, a systolic spike, or absent flow signals. However, it is important to note that TCD is not used solely to diagnose brain death but as an adjunct, since it is a clinical diagnosis [27-29].

TCD ultrasonography has not been extensively used to study PVS. Perez-Nellar et al. evaluated intracranial circulation in PVS patients using TCD. They found that, despite having standard range systolic velocities, diastolic amplitude, end-diastolic velocity, and pulsatility index were reduced in all patients. The authors concluded that the decrease in diastolic velocity and increase in pulsatility index could be related to the uncoupling of cerebral blood flow and metabolic rate, which is caused by decreased cerebral glucose consumption and oxygen uptake following extensive brain injury [30,31].

## Conclusions

Physicians and researchers in the Caribbean have used TCD to monitor various clinical conditions, such as SCD and vasospasm in SAH, and have made significant contributions to TCD-related research from the region. TCD's cost-effectiveness in monitoring cerebral blood flow in real-time makes it a valuable tool, especially in resource-constrained settings like many parts of the Caribbean. Capacity-building programs like training for healthcare providers and local TCD research networks may provide better access to TCD in more Caribbean nations.
